# Blunt Cardiac Injury in Patients With Sternal Fractures

**DOI:** 10.7759/cureus.22841

**Published:** 2022-03-04

**Authors:** Alexander A Fokin, Joanna Wycech Knight, Kai Yoshinaga, Ayesha T Abid, Robert Grady, Amaris L Alayon, Ivan Puente

**Affiliations:** 1 Surgery, Florida Atlantic University Charles E. Schmidt College of Medicine, Boca Raton, USA; 2 Trauma and Acute Care Surgery, Delray Medical Center, Delray Beach, USA; 3 Trauma and Acute Care Surgery, Broward Health Medical Center, Fort Lauderdale, USA; 4 Surgery, Florida International University, Herbert Wertheim College of Medicine, Miami, USA

**Keywords:** traumatic brain injury, troponin, echocardiography, cardiac comorbidities, blunt thoracic trauma, cardiac contusion, sternal fractures, blunt cardiac injury

## Abstract

Background

Blunt cardiac injury (BCI) is a possible consequence of sternal fractures (SF). There is a scarcity of studies addressing BCI in patients with different types of SF and with pre-existing cardiac conditions. The goal of this study was to delineate diagnostic patterns of BCI in different cohorts of SF patients.

Methods

This retrospective cohort study included 380 blunt trauma patients admitted to two level 1 trauma centers between January 2015 and March 2020 with radiologically confirmed SF. Electrocardiography, cardiac enzymes and echocardiography were evaluated for BCI diagnosis. Analyzed variables included: age, comorbidities, injury severity score, Glasgow coma score, type of SF (isolated, combined, displaced), incidence of traumatic brain injury, co-injuries, retrosternal hematoma, intensive care unit admissions, hospital lengths of stay, and mortality.

Results

In 380 SF patients there were 250 (66%) females and 130 (34%) males and the mean age was 63 years old. Electrocardiography was done in all patients, cardiac enzymes in 234 (62%) and echocardiography in 181 (48%). BCI was diagnosed in 19 (5%) of patients, all having combined SF. BCI patients had higher injury severity score (mean 18.4) and 14 (74%) had pulmonary co-injuries. Multivariable analysis confirmed pulmonary co-injuries as a statistically significant predictor of BCI (p<0.001). BCI patients compared to no BCI patients had all three tests (electrocardiography, cardiac enzymes and echocardiography) performed statistically more often (90% vs 36%, p<0.001). SF patients with pre-injury cardiac comorbidities had similar incidence of BCI as without cardiac comorbidities (5% vs 6%, p=0.6). In SF patients with traumatic brain injury, cardiac enzymes (troponin, creatine kinase) were elevated significantly more often compared to patients without traumatic brain injury (58% vs 38%, p=0.02). SF displacement or retrosternal hematoma presence were not associated with BCI. Mortality in SF patients with BCI versus without was not statistically different (16 vs 9%, p=0.4).

Conclusions

Blunt cardiac injury is rare in patients with SF. Higher degree of BCI suspicion must be applied in combined SF patients, especially those with pulmonary co-injuries. Cardiac comorbidities did not affect the rate of BCI. Echocardiography for BCI diagnosis is essential in SF patients with traumatic brain injury, as cardiac enzymes may be less informative, however is less important in* *isolated SF patients*.* Performing all three diagnostic tests in combined SF patients improves the accuracy of BCI diagnosis*.*

## Introduction

Blunt cardiac injury (BCI) is a potentially dangerous consequence of blunt thoracic trauma and it has been reported in 7% - 50% of blunt thoracic trauma patients [1-7]. The mortality rate in BCI patients ranges from 4.3% to 32% [5,7,8]. Among BCI patients, sternal fractures (SF) are diagnosed in 17% - 72% [5,8-10]. In patients with SF, BCI was reported in 2.4% - 42.6% [11-14]. As autopsy data has shown, the frequency of heart injury and SF in non-survivors of blunt trauma is even higher [15].

Variable and unspecific BCI symptoms may lead to a wide variety of interpretations and misdiagnoses. From one side, the broad array of reported data can be explained by different definitions of BCI, ranging from mild to clinically significant severity [4,7,10]. From the other side, analyzed cohorts are diverse, and may include patients with isolated SF or may contain severely injured patients after multiple trauma [10,15,16]. Certain BCI studies even exclude patients with pre-existing cardiac comorbidities (CC) [6,9]. Furthermore, discussions continue regarding the guidelines for diagnostic testing, including the sequence and combination of necessary tests to substantiate the BCI diagnosis [17]. Overall there is a scarcity of studies addressing BCI in patients with different types of SF, as well as in cohorts of patients with pre-existing cardiac conditions [14,18].

With SF on the rise, and the growing geriatric population with high incidence of CC, there is a need to develop practical recommendations for diagnosis of BCI in various cohorts of patients with SF [19].

The goal of the study was to analyze diagnostic patterns of BCI in patients with different types of SF (isolated versus combined, displaced versus non-displaced, with retrosternal hematoma versus without) and to address different clinical scenarios. Special focus was on the impact of pre-injury cardiac comorbidities on the rate of BCI and its association with cardiac interventions and mortality.

An abstract including some findings from this study was presented at the 16th Annual Academic Surgical Congress in 2021.

## Materials and methods

MetroWest Institutional Review Board issued approval 2020-105. This retrospective cohort study included 380 blunt trauma patients admitted to two urban level 1 trauma centers between January 2015 and March 2020 with radiologically confirmed SF. Patients less than 18 years old, patients with rib fractures but without SF and patients who were dead on arrival were excluded.

For diagnosis of cardiac injury, patients were assessed with various combinations of three tests: electrocardiogram (ECG), cardiac enzymes (ENZ) and echocardiogram (ECH). Analyzed variables included: age, sex, comorbidities, mechanism of injury (MOI), injury severity score (ISS), Glasgow Coma Score (GCS), type of SF (isolated, combined, displaced), presence of retrosternal hematoma, incidence of traumatic brain injury (TBI), co-injuries (pulmonary, abdominal, orthopedic), results and timing of ECG, ENZ, ECH, cardiac interventions, intensive care unit (ICU) admission, hospital LOS (HLOS) and mortality. 

Cardiac comorbidities included hypertension, coronary artery disease, atrial fibrillation, myocardial infarction, congestive heart failure, valves’ diseases and presence of a pacemaker. Pulmonary co-injuries included pulmonary contusion, hemothorax, pneumothorax and hemopneumothorax.

Abnormal ECG findings were defined as arrhythmia, AV block, bundle branch block, or ST segment deviation. Abnormal ENZ elevation was defined as values above normal laboratory thresholds (troponin >0.04 ng/mL, or creatine kinase-MB >5.0%). Abnormal ECH was defined as wall motion abnormalities, intramural hematoma, or pericardial effusion. Abnormal findings were noted in patients’ records.

For patient evaluation we adopted a previously described clinical scoring system, which was used before for stratification of patients with BCI [20]. Patients were assigned one point each for abnormality in ECG, ENZ, and ECH, for a maximum score of three [20]. Patients with one test done may have a maximum score of one point and with two tests a maximum score of two points.

Using a previously published definition, isolated sternal fractures (ISF) were defined as the only injury without any additional skeletal or visceral injury, while the rest of the SF were classified as combined sternal fractures (CSF) [18,21].

Patients ≥ 65 years old were considered geriatric.

Different cohorts of SF patients were compared: with two versus three diagnostic tests performed, with pre-existing CC versus without, with BCI versus without, and ISF versus CSF.

Statistical analysis was performed using SPSS version 23.0 (IBM Corp., Armonk, NY, USA). Comparison analyses included group characteristics and bivariate correlations. Depending on the compared groups and their distribution, Chi-squared and Fisher’s exact tests were used for categorical variables and independent samples t-tests and Mann-Whitney U test for variable means. Multinomial logistic regression analysis was performed for the mortality outcome and BCI prediction. Receiver operator characteristics area under the curve analysis was used to determine threshold values for mortality prediction variables. Statistical significance was assumed when p<0.05.

## Results

The characteristics of all patients with SF are presented in Table 1.

**Table 1:** Characteristics of Patients with Sternal Fractures

Variable	Patients with Sternal Fractures (n=380)
Age, mean years (SD)	63.3 (20.3)
Geriatric, n (%)	201 (52.9)
Sex, Female/Male, n (%)	250 (65.8) / 130 (34.2)
Comorbidities, n (%)	291 (76.6)
Cardiac Comorbidities, n (%)	180 (47.4)
Mechanism of Injury, MVC/Fall, n (%)	334 (87.9) / 46 (12.1)
Glasgow Coma Scale, mean (SD)	14.0 (2.9)
Injury Severity Score, mean (SD)	13.1 (9.5)
Traumatic Brain Injury, n (%)	49 (12.9)
Spine Co-Injury, n (%)	146 (38.4)
Abdominal Co-Injury, n (%)	40 (10.5)
Orthopedic Co-Injury, n (%)	134 (35.3)
Pulmonary Co-Injury, n (%)	133 (35.0)
Displaced Sternal Fracture, n (%)	111 (29.2)
Retrosternal Hematoma, n (%)	106 (27.9)
Combined Sternal Fracture, n (%)	312 (82.1)
Blunt Cardiac Injury, n (%)	19 (5.0)
Cardiac Procedure, n (%)	5 (1.3)
ICU Admission, n (%)	205 (53.9)
Hospital Length of Stay, mean days (SD)	9.4 (13.8)
Mortality, n (%)	29 (7.6)

Mean age of SF patients was 63 years old, with an average ISS of 13.1 and main MOI being motor vehicle collisions (MVC).

There were a total of 19 (5.0%) SF patients diagnosed with BCI, all due to MVC and all with CSF. BCI and no BCI groups had comparable rates of geriatric patients (63.2% vs 49.5%, p=0.3), cardiac comorbidities (52.6% vs 45.1%, p=0.6), but BCI patients had significantly higher rate of pulmonary co-injury (73.7% vs 40.6%, p=0.007). In 17 out of the 19 BCI patients (89.5%) all three (ECG, ENZ, ECH) diagnostic tests were done, while among no BCI patients all three tests (ECG, ENZ, ECH) were done in 130 (36.0%, p<0.001). Five BCI patients with combined SF required urgent cardiac interventions based on clinical symptoms: one for an aortic valve repair, two for a ventricle wall laceration and an epicardial coronary artery injury, and two for an aortic aneurysm dissection. Mortality was not statistically different between BCI and no BCI groups (15.8% vs 8.9%, p=0.4). Multivariable analysis for BCI predictors showed the presence of pulmonary co-injuries as a statistically significant factor (p<0.001).

**Figure 1:**
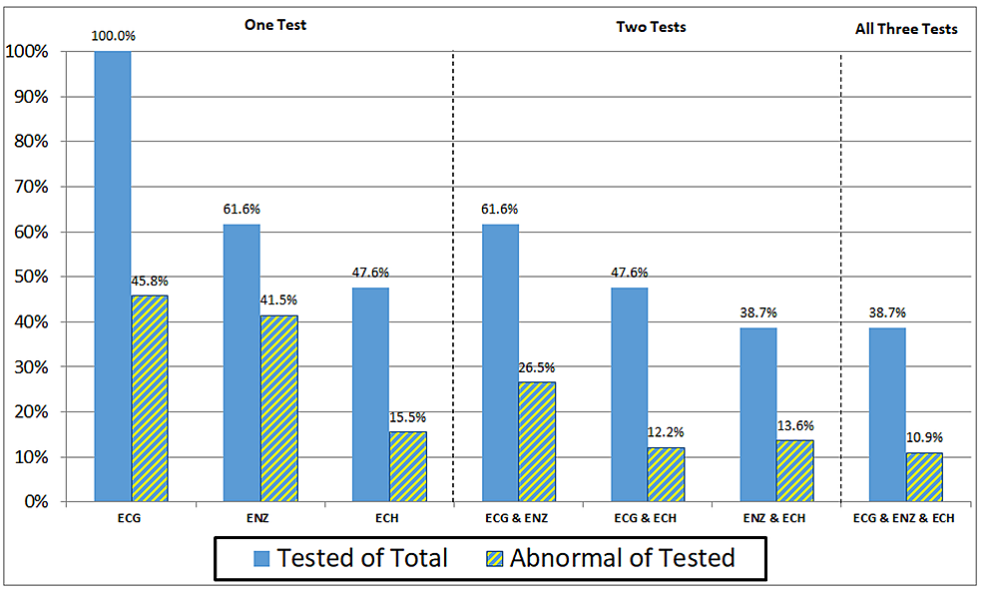
Rates of Tests Performed and Rates of Abnormal Tests in All Patients with Sternal Fractures ECG - Electrocardiography; ENZ - Cardiac Enzymes; ECH - Echocardiography

The frequency of performed diagnostic tests in different combinations and the rate of abnormal tests are presented in Figure 1.

ENZ and ECH were never done without a preceding ECG. Mean time from the emergency department admission to an ECG was 3.1 hours, to ENZ was 15.2 hours and to ECH was 43.3 hours.

ECH was done statistically more often (p=0.001) when ENZ were elevated (73 out of 97 patients or 75.3%) compared to when ENZ were normal (74 out of 137 patients or 54.0%). ECH was also abnormal statistically significantly more often (p=0.002) when ENZ were elevated (20 out of 73 tested patients or 27.4%) compared to when ENZ were normal (six out of 74 tested patients or 8.1%).

An ECG alone was performed in 112 (29.5%) patients, two tests (ECG plus ENZ or ECG plus ECH) in 121 (31.8%) patients, and all three tests in 147 (38.7%) patients. A comparison of patients with two versus three diagnostic tests performed is presented in Table 2. 

**Table 2:** Comparison of Patients with Sternal Fracture with Two versus Three Diagnostic Tests Performed *-denotes a statistically significant difference; MVC – Motor vehicle collision; ECG – Electrocardiography; ENZ – Enzymes; ECH – Echocardiography

Variable	Two Tests (n=121)	Three Tests (n=147)	P value
Age, mean years (SD)	65.1 (18.4)	66.9 (18.8)	0.4
Geriatric, n (%)	71 (58.7)	88 (59.9)	0.8
Sex, Female/Male, n (%)	82 (67.8) / 39 (32.2)	93 (63.3) / 54 (36.7)	0.4
Comorbidities, n (%)	94 (77.7)	120 (81.6)	0.4
Cardiac Comorbidities, n (%)	57 (47.1)	88 (59.9)	0.04*
Mechanism of Injury, MVC/Fall, n (%)	106 (87.6) / 15 (12.4)	130 (88.4) / 17 (11.6)	0.8
Glasgow Coma Scale, mean (SD)	14.0 (2.8)	13.8 (3.1)	0.6
Injury Severity Score, mean (SD)	12.5 (9.9)	14.0 (10.2)	0.2
Traumatic Brain Injury, n (%)	10 (8.3)	24 (16.3)	0.04*
Spine Co-Injury, n (%)	44 (36.4)	55 (37.4)	0.9
Abdominal Co-Injury, n (%)	7 (5.8)	25 (17.0)	0.005*
Orthopedic Co-Injury, n (%)	45 (37.2)	52 (35.4)	0.8
Pulmonary Co-Injury, n (%)	36 (29.8)	57 (38.8)	0.1
Displaced Sternal Fracture, n (%)	37 (30.6)	44 (29.9)	0.9
Retrosternal Hematoma, n (%)	32 (26.4)	46 (31.3)	0.4
Combined Sternal Fracture, n (%)	96 (79.3)	119 (81.0)	0.7
ECG done, n (%)	121 (100.0)	147 (100.0)	1.0
ECG abnormal, n (%)	59 (48.8)	91 (61.9)	0.03*
ENZ done, n (%)	87 (71.9)	147 (100.0)	<0.001*
ENZ abnormal, n (%)	24 (27.6)	73 (49.7)	0.001*
ECH done, n (%)	34 (28.1)	147 (100.0)	<0.001*
ECH abnormal, n (%)	2 (5.9)	26 (17.7)	0.1
ECG & ENZ done, n (%)	87 (71.9)	147 (100.0)	<0.001*
ECG & ENZ both abnormal, n (%)	13 (14.9)	49 (33.3)	0.002*
ECG & ECH done, n (%)	34 (28.1)	147 (100.0)	<0.001*
ECG & ECH both abnormal, n (%)	1 (2.9)	21 (14.3)	0.07
ENZ & ECH done, n (%)	0 (0.0)	147 (100.0)	<0.001*
ENZ & ECH both abnormal, n (%)	-	20 (13.6)	-
ECG & ENZ & ECH done, n (%)	0 (0.0)	147 (100.0)	<0.001*
ECG & ENZ & ECH all abnormal, n (%)	-	16 (10.9)	-
Blunt Cardiac Injury, n (%)	2 (1.7)	17 (11.6)	0.002*
Intensive Care Unit Admission, n (%)	58 (47.9)	103 (70.1)	<0.001*
Hospital Length of Stay, mean days (SD)	7.0 (9.6)	11.8 (13.6)	0.001*
Mortality, n (%)	8 (6.6)	18 (12.2)	0.1

**Figure 2:**
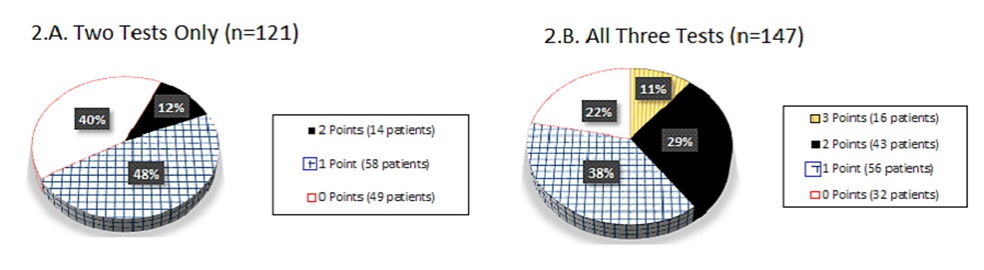
A. Distribution by Scoring Points of Patients with Two Tests Performed in Any Combination; B. Distribution by Scoring Points of Patients with Three Tests Performed

Stratification of patients with two or three tests by clinical scoring points for abnormality in ECG, ENZ and ECH is presented in pie graphs (Figure 2).

The maximum number of clinical scoring points was significantly higher in BCI patients compared to no BCI patients (eight versus 22 patients, or 42.1% versus 8.8%, p<0.001).

In additional analysis, in 61 patients with TBI and/or GCS ≤8, ENZ were elevated (23 out of 40 or 57.5%) statistically significantly more often (p=0.02) compared to 319 patients without TBI and/or GCS >8 (74 out of 194 or 38.1%).

Comparison of patients with versus without cardiac comorbidities is presented in Table 3.

**Table 3:** Comparison of Patients with Sternal Fractures with and without Cardiac Comorbidities *-denotes a statistically significant difference; MVC – Motor vehicle collision; ECG – Electrocardiography; ENZ – Enzymes; ECH – Echocardiography

Variable	No Cardiac Comorbidity (n=200)	Cardiac Comorbidity (n=180)	P value
Age, mean (SD)	53.1 (20.4)	74.7 (12.7)	<0.001*
Geriatric, n (%)	62 (31.0)	139 (77.2)	<0.001*
Sex, Female/Male, n (%)	133 (66.5) / 67 (33.5)	117 (65.0) / 63 (35.0)	0.8
Comorbidities, n (%)	111 (55.5)	180 (100.0)	<0.001*
Mechanism of Injury, MVC/Fall, n (%)	178 (89.0) / 22 (11.0)	156 (86.7) / 24 (13.3)	0.4
Glasgow Coma Scale, mean (SD)	13.7 (3.4)	14.3 (2.2)	0.03*
Injury Severity Score, mean (SD)	13.8 (9.5)	12.3 (9.4)	0.1
Traumatic Brain Injury, n (%)	31 (15.5)	18 (10.0)	0.1
Spine Co-Injury, n (%)	75 (37.5)	71 (39.4)	0.7
Abdominal Co-Injury, n (%)	26 (13.0)	14 (7.8)	0.1
Orthopedic Co-Injury, n (%)	77 (38.5)	57 (31.7)	0.2
Pulmonary Co-Injury, n (%)	74 (37.0)	59 (32.8)	0.4
Displaced Sternal Fracture, n (%)	56 (28.0)	55 (30.6)	0.6
Retrosternal Hematoma, n (%)	49 (24.5)	57 (31.7)	0.1
Combined Sternal Fracture, n (%)	170 (85.0)	142 (78.9)	0.2
ECG done, n (%)	200 (100.0)	180 (100.0)	1.0
ECG abnormal, n (%)	73 (36.5)	101 (56.1)	<0.001*
ENZ done, n (%)	106 (53.0)	128 (71.1)	<0.001*
ENZ abnormal, n (%)	35 (33.0)	62 (48.4)	0.02*
ECH done, n (%)	76 (38.0)	105 (58.3)	<0.001*
ECH abnormal, n (%)	10 (13.2)	18 (17.15)	0.5
ECG & ENZ done, n (%)	106 (53.0)	128 (71.1)	<0.001*
ECG & ENZ both abnormal, n (%)	21 (19.8)	41 (32.0)	0.04*
ECG & ECH done, n (%)	76 (38.0)	105 (58.3)	<0.001*
ECG & ECH both abnormal, n (%)	7 (9.2)	15 (14.3)	0.3
ENZ & ECH done, n (%)	59 (29.5)	88 (48.9)	<0.001*
ENZ & ECH both abnormal, n (%)	6 (10.2)	14 (15.9)	0.3
ECG & ENZ & ECH done, n (%)	59 (29.5)	88 (48.9)	<0.001*
ECG & ENZ & ECH all abnormal, n (%)	4 (6.8)	12 (13.6)	0.2
Blunt Cardiac Injury, n (%)	9 (4.5)	10 (5.6)	0.6
Intensive Care Unit Admission, n (%)	97 (48.5)	108 (60.0)	0.03*
Hospital Length of Stay, mean days (SD)	10.1 (16.3)	8.6 (10.4)	0.3
Mortality, n (%)	10 (5.0)	19 (10.6)	0.04*

BCI was diagnosed comparably often in both cohorts. When hypertension (being too common of a condition with a wide range of clinical presentations) was removed as a CC to accentuate the severity of CC, 306 patients without CC had a 4.9% BCI rate compared with a 5.4% BCI rate in 74 patients with CC (p=0.8).

The rate of BCI in patients with displaced SF was similar to patients with not displaced SF (4.5% vs. 5.2%, p=0.8). There was also no difference in rates of BCI in patients with retrosternal hematoma compared to patients without a retrosternal hematoma (3.8% vs. 5.5%, p=0.5).

Comparison of isolated versus combined SF patients is presented in Table 4.

**Table 4:** Comparison of Patients with Isolated Sternal Fracture versus Combined Sternal Fracture *-denotes a statistically significant difference; SF – Sternal Fracture; MVC – Motor vehicle collision; ECG – Electrocardiography; ENZ – Enzymes; ECH – Echocardiography

Variable	Isolated SF (n=68)	Combined SF (n=312)	P value
Age, mean years (SD)	70.1 (17.0)	61.9 (20.6)	0.001*
Geriatric, n (%)	44 (64.7)	157 (50.3)	0.03*
Sex, Female/Male, n (%)	51 (75.0) / 17 (25.0)	199 (63.8) / 113 (36.2)	0.1
Comorbidities, n (%)	56 (82.4)	235 (75.3)	0.2
Cardiac Comorbidities, n (%)	38 (55.9)	142 (45.5)	0.1
Mechanism of Injury, MVC/Fall, n (%)	58 (85.3) / 10 (14.7)	276 (88.5) / 36 (11.5)	0.5
Glasgow Coma Scale, mean (SD)	14.9 (0.3)	13.8 (3.2)	<0.001*
Injury Severity Score, mean (SD)	4.5 (1.2)	14.9 (9.5)	<0.001*
Traumatic Brain Injury, n (%)	0 (0.0)	49 (15.7)	<0.001*
Spine Co-Injury, n (%)	0 (0.0)	146 (46.8)	<0.001*
Abdominal Co-Injury, n (%)	0 (0.0)	40 (12.8)	0.002*
Orthopedic Co-Injury, n (%)	0 (0.0)	134 (42.9)	<0.001*
Pulmonary Co-Injury, n (%)	0 (0.0)	133 (42.6)	<0.001*
Displaced Sternal Fracture, n (%)	24 (35.3)	87 (27.9)	0.2
Retrosternal Hematoma, n (%)	23 (33.8)	83 (26.6)	0.2
ECG done, n (%)	68 (100.0)	312 (100.0)	1.0
ECG abnormal, n (%)	34 (50.0)	140 (44.9)	0.4
ENZ done, n (%)	47 (69.1)	187 (59.9)	0.2
ENZ abnormal, n (%)	9 (19.1)	88 (47.1)	0.001*
ECH done, n (%)	34 (50.0)	147 (47.1)	0.7
ECH abnormal, n (%)	0 (0.0)	28 (19.0)	0.006*
ECG & ENZ done, n (%)	47 (69.1)	187 (59.9)	0.2
ECG & ENZ both abnormal, n (%)	4 (8.5)	58 (31.0)	0.002*
ECG & ECH done, n (%)	34 (50.0)	147 (47.4)	0.7
ECG & ECH both abnormal, n (%)	0 (0.0)	22 (15.0)	0.02*
ENZ & ECH done, n (%)	28 (41.2)	119 (38.1)	0.6
ENZ & ECH both abnormal, n (%)	0 (0.0)	20 (16.8)	0.02*
ECG & ENZ & ECH done, n (%)	28 (41.2)	120 (38.5)	0.6
ECG & ENZ & ECH all abnormal, n (%)	0 (0.0)	16 (13.4)	0.04*
Blunt Cardiac Injury, n (%)	0 (0.0)	19 (6.1)	0.04*
Intensive Care Unit Admission, n (%)	16 (23.5)	189 (60.6)	<0.001*
Hospital Length of Stay, mean days (SD)	2.8 (2.8)	10.8 (14.8)	<0.001*
Mortality, n (%)	0 (0.0)	29 (9.3)	0.009*

As expected, ICU admissions and HLOS were significantly higher in the CSF group. There was no mortality in ISF and the mortality in CSF was 9.3%.

Overall mortality among SF patients was 7.6%. Analysis of all 29 mortality cases revealed that all expired patients had CSF, 22 were geriatric, 19 had CC and 12 had brain injury, however, only three had BCI. Only in one BCI patient was mortality directly related to BCI. Multivariable and receiver operator characteristics analyses of 380 SF patients revealed that age (>70), GCS (<13) and CSF were significant predictors of mortality.

## Discussion

Over 63 months there were 380 SF patients admitted to two urban, level 1 trauma centers, half of them were geriatric, approximately half had CC, and approximately one-fifth had ISF, while the rest had CSF.

In our patients with SF, the overall diagnosed rate of BCI was 5.0% and is close to the reported BCI incidence of 2.5% - 3.9% from several large queries of the National Trauma Data Bank [12-13].

The most common diagnostic test was an ECG done in all patients, followed by ENZ done in approximately two-thirds of patients, and ECH done in approximately half of patients. The overall frequency of ECH evaluation in our study was 47.6% and was the highest among BCI patients with 89.5%, followed by patients with CC at 58.3%. In comparison, ECH evaluation was reported in 76% of patients with BCI by Hammer et al. [20], in 45% by Skinner et al. [7], but only in 7% of ISF patients by Dua et al. [16]. 

Among SF patients, an abnormal ECH had the lowest prevalence out of all diagnostic tests. ECH was done statistically more often when ENZ were elevated compared to when ENZ were normal and it was also abnormal significantly more often when ENZ were elevated. These findings are in compliance with previously published observations that abnormal ECH is detected only after ENZ elevation reaches a certain threshold [6,22-24]. A two-day delay to ECH evaluation in our patients may have affected the findings, as medication given and recovery progress may have resulted in clinical improvement.

We also looked for differences in patient characteristics between those who received two versus three diagnostic tests, and if that led to changes in the diagnosis of BCI. Two or three diagnostic tests were done in approximately one-third of patients. Patients who received all three tests were more likely to have CC, TBI and abdominal co-injuries. BCI was diagnosed seven times more often in the three tests group and it was statistically significant.

Among BCI patients, the maximal number of scoring points was observed almost five times more compared to patients with no BCI and the difference was statistically significant. Our rate of 41.2% for maximal scoring points/all three abnormal tests in BCI patients was similar to that of 36.4% by Hammer et al. [20] among their SF patients, and is in agreement with findings that not all BCI patients had all three tests abnormal. If the definition of BCI included having all three tests abnormal, the addition of these patients would increase the BCI rate in our study from 5.0% to 7.4%. 

We agree with the opinion that no single test could serve as a gold standard for myocardial contusion [17,25]. The questions of what constitutes BCI, what tests are the most accurate for BCI diagnosis, or what combination of tests is the best for certain cohorts of blunt thoracic trauma patients, remain in discussion. Conceivably, the definition of BCI could also include the timing of an optimal diagnostic window, as a very early ENZ test may show normal values, and similarly the results of a very late ECH can be inviolate since BCI symptoms may already be resolved with time and/or medication.

In our study, pulmonary co-injuries, including pulmonary contusion, hemothorax, pneumothorax and hemopneumothorax, were the only statistically significant predictor for BCI diagnosis. These findings are consistent with a recent National Trauma Data Bank analysis by Grigorian et al. [8] who found that in blunt trauma a hemopneumothorax is the strongest predictor for BCI, followed by an SF. Mortality of 16% in our BCI patients was also similar to mortality of 19% reported by Grigorian et al. in blunt trauma patients with BCI [8].

One of the less addressed topics is the effect of pre-existing cardiac comorbidities on the diagnosis of BCI in patients with SF. In our study, patients with CC were similarly injured, with identical MOI and isolated/combined SF type distribution, but were significantly older than patients without CC. ICU admissions and mortality were significantly higher in patients with CC, which can be attributed to the substantially older age, more comorbidities. CC patients were more likely to get all three diagnostic tests in all combinations. ECG and ENZ separately and in combination were abnormal statistically more often in the CC group compared to the group without CC, but frequency of abnormal ECH was not statistically different. BCI was diagnosed comparably often in both groups (around 5%), and even when we considered a more strict definition of CC and removed hypertension from the list, we still found no difference in the BCI prevalence. Therefore, even with the higher level of caution and care (increased number of diagnostic tests and ICU admissions), having CC did not result in a higher rate of BCI diagnosis. Rashid et al. [3] reported that the two SF patients with co-existing cardiac diseases had no cardiac problems from the sternal fracture. In comparison, Brookes et al. [18] stated that SF patients over 65 years old with pre-existing ischemic heart disease or on digoxin therapy, are at risk for cardiac event and require monitoring.

There is a variability of data regarding the prevalence of BCI in ISF, as well as the diagnostic value of abnormal tests in polytrauma patients. Evidently, CSF patients in comparison to ISF patients were more severely injured. Furthermore, all of the patients diagnosed with BCI were in the CSF group. Our data are in contradiction with the observations of De Waele et al. [26], who reported a high BCI incidence of 32% in patients with ISF. Surprisingly, all three BCI diagnostic tests, in any combinations, were performed comparably often in ISF and CSF patients. In CSF patients ENZ were elevated more often. However, the ENZ elevation has also been documented in TBI patients as well as in other non-cardiac conditions [23,27-30]. In our SF patients with TBI/low GCS, enzymes were elevated statistically significantly more often compared to SF patients without TBI/low GCS. These observations indicate that in SF patients with suspected BCI, who also have a TBI, assessment of enzymes may be less informative, consequently making an ECG and ECH test combination more appropriate. ECH was not affected in ISF patients and was only abnormal in CSF patients, hence it is not indicated in patients with ISF. Therefore, in certain subgroups of SF patients, the indicative accuracy of the applied tests for BCI diagnosis may vary and for that reason different diagnostic strategies should be considered.

SF displacement was present in approximately one third of patients in different cohorts, was never a factor in any of our comparisons, and did not contribute to a higher rate of BCI. These findings are in line with conclusions by Heidelberg et al. [14] that there is no significant association between the depth of SF displacement and BCI. Our data are in contradiction with the observations of De Waele et al. [26], who reported statistically higher BCI incidence in cases of displaced SF.

Retrosternal hematoma was present in one fourth of our patients and its incidence was consistently similar and not significantly different between all compared groups. Our findings are in accordance with the conclusion by Heidelberg et al. [14] that retrosternal hematoma is not associated with diagnosis of BCI. Rashid et al. [3] also found no difference in the incidence of associated injuries, including cardiac injuries in SF patients with or without retrosternal hematoma. 

When heart is bruised, and blood effused, the term contused, is vast misused, but with data inclusion, and science diffusion, the riling confusion, is changed to conclusion, with no delusion.

**Limitations

The retrospective nature of the study with known deficiencies of prerecorded data and diagnostic interpretations. Cardiac events after blunt chest trauma in patients without SF were not analyzed. The degree of SF displacement was not evaluated; just the presence or absence were noted. Dichotomized stratification of tests included only normal versus abnormal comparison without analyses of ranges of absolute values.

## Conclusions

Blunt cardiac injury is a rare condition and is diagnosed in 5.0% of trauma patients with sternal fractures. Pre-injury cardiac comorbidities did not affect the rate of BCI diagnosis. SF displacement and the presence of retrosternal hematoma were not associated with BCI. BCI was diagnosed only in combined SF patients; therefore, a higher degree of suspicion must be applied in combined SF patients, especially in the presence of pulmonary co-injuries, which were found to be an independent risk factor for BCI. In isolated SF patients, echocardiography is less essential. In SF patients with TBI, ECH is recommended, as ENZ values may be less informative. Performing all three diagnostic tests in combined SF patients improves the accuracy of BCI diagnosis.
